# RNA-Seq analysis reveals potential regulators of programmed cell death and leaf remodelling in lace plant (*Aponogeton madagascariensis*)

**DOI:** 10.1186/s12870-021-03066-7

**Published:** 2021-08-13

**Authors:** Nathan M. Rowarth, Bruce A. Curtis, Anthony L. Einfeldt, John M. Archibald, Christian R. Lacroix, Arunika H. L. A. N. Gunawardena

**Affiliations:** 1 Department of Biology, Dalhousie University, Halifax, NS Canada; 2 Department of Biochemistry & Molecular Biology, Dalhousie University, Halifax, NS Canada; 3 Department of Biology, University of Prince Edward Island, Charlottetown, PEI Canada

**Keywords:** Anthocyanin, Developmental PCD, RNA sequencing, Laser capture microdissection, Transcriptomes

## Abstract

**Background:**

The lace plant (*Aponogeton madagascariensis*) is an aquatic monocot that develops leaves with uniquely formed perforations through the use of a developmentally regulated process called programmed cell death (PCD). The process of perforation formation in lace plant leaves is subdivided into several developmental stages: pre-perforation, window, perforation formation, perforation expansion and mature. The first three emerging “imperforate leaves” do not form perforations, while all subsequent leaves form perforations via developmentally regulated PCD. PCD is active in cells called “PCD cells” that do not retain the antioxidant anthocyanin in spaces called areoles framed by the leaf veins of window stage leaves. Cells near the veins called “NPCD cells” retain a red pigmentation from anthocyanin and do not undergo PCD. While the cellular changes that occur during PCD are well studied, the gene expression patterns underlying these changes and driving PCD during leaf morphogenesis are mostly unknown. We sought to characterize differentially expressed genes (DEGs) that mediate lace plant leaf remodelling and PCD. This was achieved performing gene expression analysis using transcriptomics and comparing DEGs among different stages of leaf development, and between NPCD and PCD cells isolated by laser capture microdissection.

**Results:**

Transcriptomes were sequenced from imperforate, pre-perforation, window, and mature leaf stages, as well as PCD and NPCD cells isolated from window stage leaves. Differential expression analysis of the data revealed distinct gene expression profiles: pre-perforation and window stage leaves were characterized by higher expression of genes involved in anthocyanin biosynthesis, plant proteases, expansins, and autophagy-related genes. Mature and imperforate leaves upregulated genes associated with chlorophyll development, photosynthesis, and negative regulators of PCD. PCD cells were found to have a higher expression of genes involved with ethylene biosynthesis, brassinosteroid biosynthesis, and hydrolase activity whereas NPCD cells possessed higher expression of auxin transport, auxin signalling, aspartyl proteases, cysteine protease, Bag5, and anthocyanin biosynthesis enzymes.

**Conclusions:**

RNA sequencing was used to generate a de novo transcriptome for *A. madagascariensis* leaves and revealed numerous DEGs potentially involved in PCD and leaf remodelling. The data generated from this investigation will be useful for future experiments on lace plant leaf development and PCD in *planta*.

**Supplementary Information:**

The online version contains supplementary material available at 10.1186/s12870-021-03066-7.

## Background

### Programmed cell death

Programmed cell death (PCD) is an essential developmental process that ensures the removal of cells, for tissue remodelling or in response to environmentally induced stress [1–4]. Plant developmental PCD is controlled by internal and external signals resulting in the organization of developing tissues [5–7]. Examples include embryonic suspensor deletion [8], aerenchyma formation [9], and xylem differentiation [10, 11]. Due to the experimental challenges associated with physically separating PCD destined cells spatially and temporally in plant model systems, there is presently little known about the genetic mechanisms that control developmental PCD. Plant systems with adjacent regions of differing cells fates arising from perforation formation during leaf morphogenesis can provide unique insight into the PCD process [12].

### The lace plant model system

The formation of leaf perforations during development is unique and has been studied in plant families such as Araceae and Aponogetonaceae [12]. *Monstera obliqua*, *M. deliciosa*, and *Aponogeton madagascariensis* utilize PCD to dismantle and clear designated cells during early shoot development, creating perforations in the leaf blade.

The lace plant *A. madagascariensis* is an aquatic monocot that has recently emerged as a model system for studying PCD, due to the accessibility and predictability of PCD in developing leaves. Lace plant leaves are also thin and translucent, making them ideal for live-cell microscopy. Finally, the sterile propagation of whole lace plants in axenic environments create opportunities for pharmacological studies [3, 12].

**Fig. 1:**
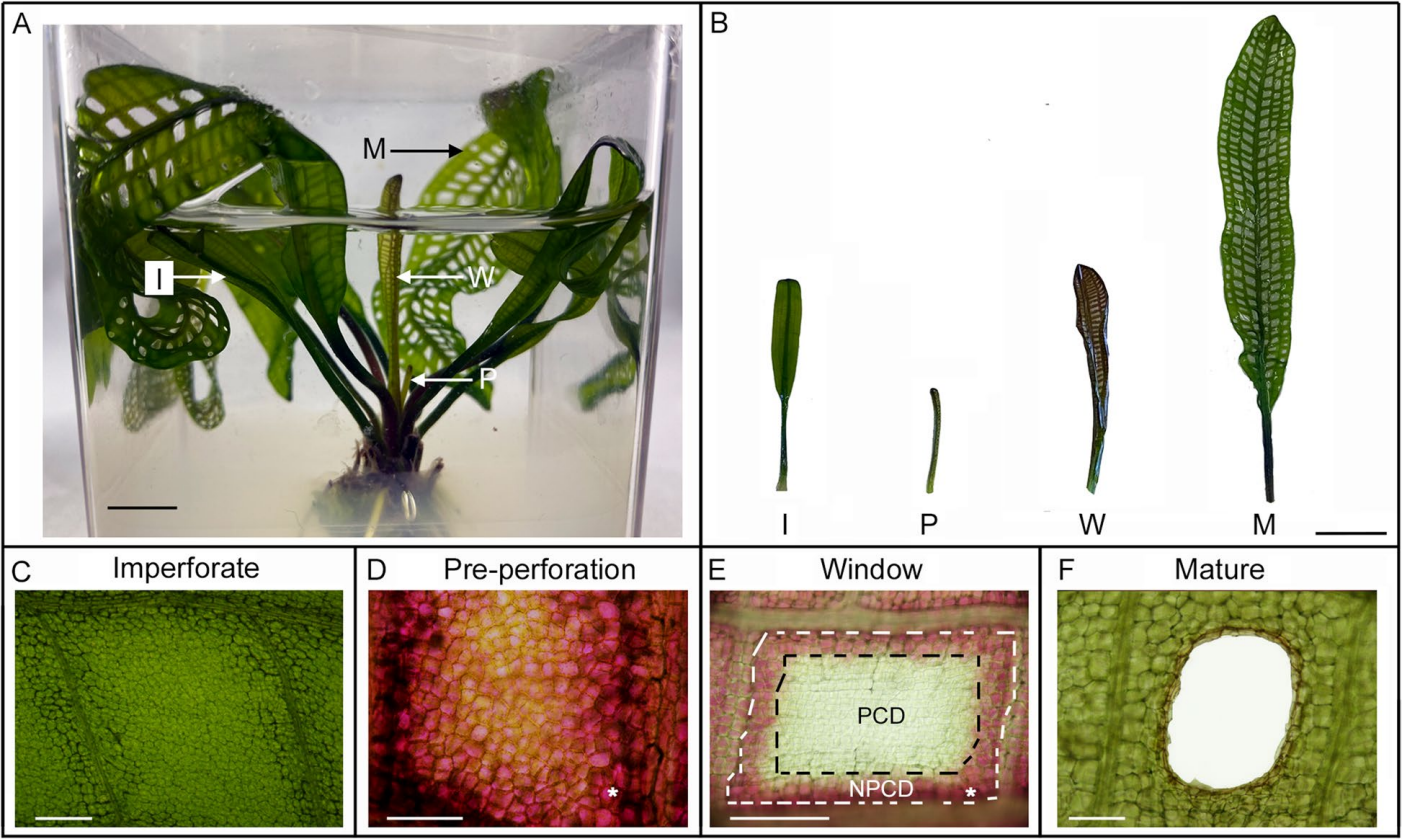
The lace plant programmed cell death (PCD) model system. **A**-**B** Lace plant grown in axenic Magenta box culture with the pre-perforation stage (P), window stage (W), mature stage (M), and imperforate leaves (I). **C** Imperforate leaves emerge from the corm lacking anthocyanin and forming areoles with no perforations. **D** Successive pre-perforation stage leaves emerge from the corm with anthocyanin pigmentation (indicated by *asterisks*). **E** PCD can be seen actively occurring in the window stage of development. Between longitudinal and transverse veins, in spaces known as areoles, a gradient of cell death can be observed. Non-PCD cells (NPCD; bounded by *white dashed lines*) cells persist beyond maturity. PCD cells (bounded by *black dashed lines*) have lost their anthocyanins, are translucent and on the verge of death. PCD and perforation formation is complete in mature stage leaves (**F**) and anthocyanin pigmentation is visibly reduced, and homeostasis for NPCD cells is reached. Scale bars: A = 1 cm; B = 2 cm; C = 200 μm

The lace plant generates leaves with a perforated morphology, wherein specific cells bounded within the vasculature are removed via developmentally regulated PCD (Fig. 1A). Leaves of the lace plant emerge in a heteroblastic series from an underground corm, and while the first 3–4 leaves (termed imperforate leaves, Fig. 1B, C) to emerge do not form perforations at all during development, all successive emerging leaves become perforated (termed adult leaves). Early adult leaves in the “pre-perforation stage” (Fig. 1B, D) emerge from the corm in a furled form, with an abundance of anthocyanin and complete vein pattern [12, 13]. As pre-perforation leaves unfurl they transition to the “window stage” (Fig. 1B, E). Window stage is reached when cells in the central portion of areoles located between longitudinal and transverse veins undergo PCD, losing their anthocyanin and chlorophyll pigmentation (“PCD cells”, Fig. 1E). Cells proximal to the veins retain both anthocyanin and chlorophyll pigmentation and do not undergo PCD; and are therefore called “non-PCD cells” (NPCD cells, Fig. 1E). The perforations increase in size until halting 4–5 cell layers from the veins. Once perforation formation is nearly completed, the mesophyll cells at the NPCD cell edge of perforations transdifferentiate into epidermal cells. The leaves reach the mature stage once perforation expansion halts and NPCD cells achieve homeostasis (Fig. 1B, F).

### Lace plant PCD mechanism

The visible gradient of PCD within the areoles of window stage leaves represents a powerful model system for studying cellular changes across this gradient during developmental PCD [12]. The cellular changes that occur across the gradient of PCD in the lace plant have been well characterized, and some of the early events of lace plant PCD include the reorganization of the actin cytoskeleton, chloroplast ring formation around the nucleus, mitochondrial aggregation, and increased number of transvacuolar strands [12]. The manner in which organelles are taken up into the vacuole in membrane-bound vesicles suggest the involvement of autophagy [14]. Further examples of cellular changes include loss of mitochondrial membrane potential, DNA fragmentation, and activation of caspase-like proteases before vacuolar collapse, plasma membrane shrinkage and cell wall degradation [15, 16].

In spite of the well characterized progression of PCD, little is known about the molecular mechanisms that control lace plant PCD regulation and execution [17, 18], in part due to a lack of molecular information for the Aponogetonaceae family. The advancement of comparative RNA sequencing (RNA-Seq) analysis between PCD and NPCD-like cells in other plant models has helped characterize differentially expressed genes (DEGs) that resemble PCD regulators. RNA-Seq analysis of separated embryonal mass and suspensor cells of *Picea abies* has shown that a spruce homolog of bax inhibitor-1 transcript is upregulated in early PCD suspensor cells and plays a role in regulating vacuolar cell death in suspensor cells [19]. Moreover, RNA-Seq analysis of early and late cavern forming leaf aerenchyma cells of *Typha angustifolia* revealed expansins, calmodulin-like proteins and pectinases transcripts that were directly related to lysigenous aerenchyma induction [20].

To date there is only one transcriptome study, Rantong et al. (2016) [18], which investigated lace plant leaf stages using complementary DNA-amplified fragment length polymorphism (cDNA-AFLP). This study identified 79 annotated DEGs which are involved in processes such as photosynthesis, stress responses, pathogen defence, and PCD. Importantly, their results suggested that ubiquitin-proteosome machinery may be involved in lace plant PCD. However, as cDNA-AFLP captures only a fraction of the transcriptome, how expression patterns across the entire *A. madagascariensis* genome change during PCD remains unknown.

In this study, we used high-throughput RNA-Seq to compare global transcriptome expression profiles of different stages of lace plant development as well as PCD and NPCD cells. We separated PCD and NPCD cells within window stage leaves by laser capture microdissection, allowing us to identify DEGs that may be involved in PCD and survival. We also identified, through differential expression analyses of RNA-Seq data, genes highly expressed in PCD and NPCD cells that are potential PCD inductors, executors and/or regulators. To identify key regulators of lace plant leaf remodelling, we additionally tested for genes that are highly expressed among perforating and non-perforating leaves.

Our objectives are to identify and compare DEGs among different leaf developmental stages and between PCD and NPCD cells. We hypothesize that imperforate and mature leaves have significantly higher levels of expression of genes involved in photosynthesis and negative regulation of PCD while pre-perforation and window leaves will have significantly higher levels of expression of genes responsible for anthocyanin biosynthesis, caspase-like activity, cell wall organization, and pro-PCD regulation.

## Results and discussion

### RNA-Seq data overview

To identify potential regulators of lace plant developmental PCD and leaf remodelling, we generated a novel lace plant transcriptome and identified DEGs in comparisons of pre-perforation, window, mature and imperforate leaf stages, and NPCD and PCD cell types using RNA-Seq. Eighteen paired-end RNA-Seq libraries were generated from three biological replicates of each imperforate, pre-perforation, window, mature leaf stages, NPCD cells and PCD cells. The Illumina (San Diego, CA, USA) NovaSeq6000 sequencing platform was used for paired-end sequencing at Génome Québec (Montréal, QC, Canada), with a 100 bp read length. A total of 1,320,261,351 reads were generated, and data for individual biological libraries were deposited to the NCBI SRA database with the following SRA accession IDs: SRR10524134-SR10524151 and BIOPROJECTID: PRJNA591467. After read filtering, 1,288,318,561 reads remained and 1,102,201,639 reads aligned concordantly (Additional file 1).

**Fig. 2:**
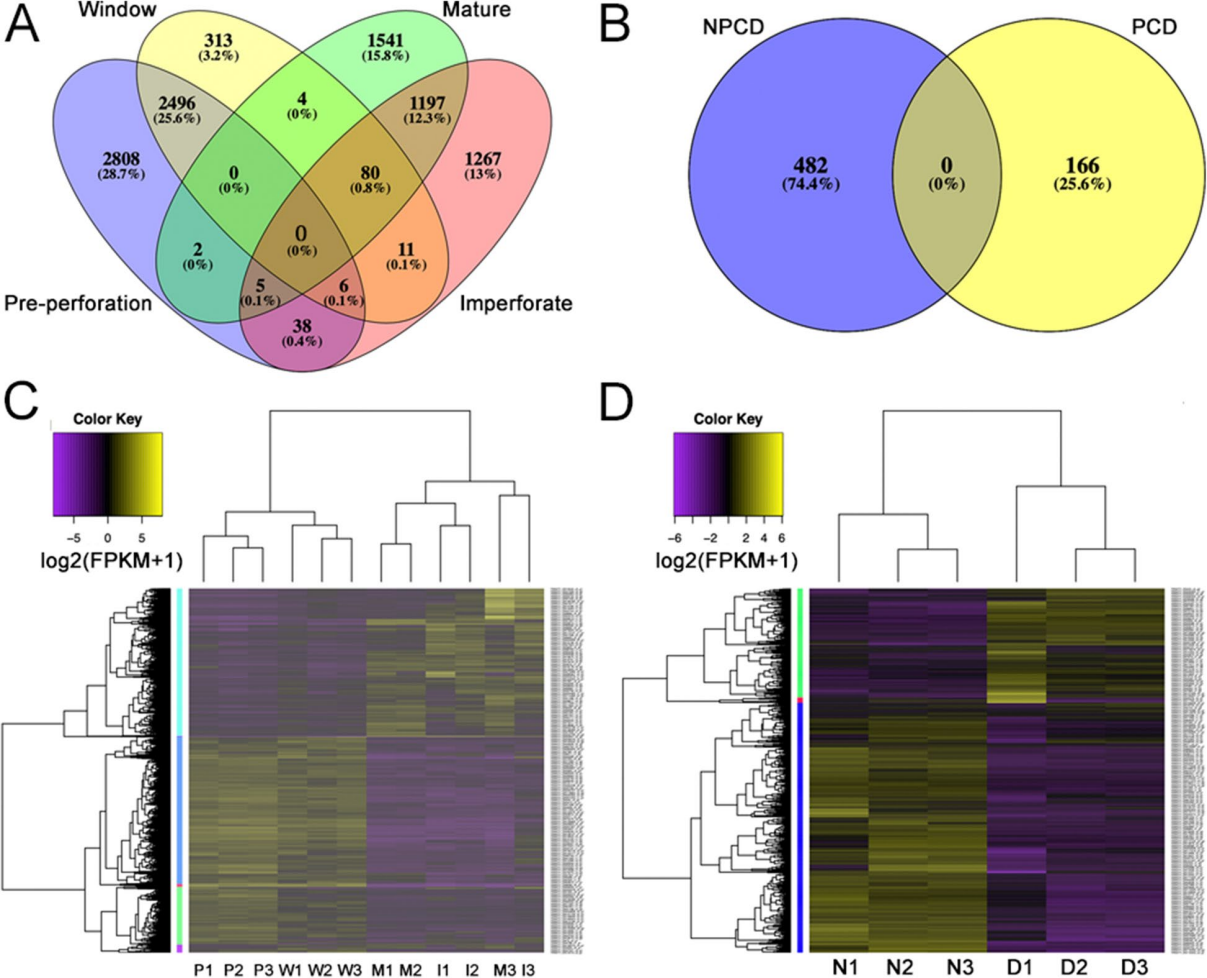
Overview of differentially expressed genes in lace plant leaves and cell types. Venn diagram showing the number of mutual and exclusive differentially expressed genes (DEGs) between lace plant leaf stages (**A**) and between NPCD and PCD cells (**B**); fold change > 2.0, *P* < 0.01. Heatmaps of gene expression levels of DEGs plotted as log2(FPKM + 1) for indicated samples and biological replicates for leaf stage comparison (**C**) and comparison of NPCD and PCD cells (**D**). Low and high gene expression levels are shown in purple and yellow, respectively. Below heatmaps, biological replicates 1–3 are indicated by *P*, pre-perforation; *W*, window; *M*, mature; and *I*, imperforate. Supporting data for gene expression found in Additional file 3 and 5

We assembled 908,119 transcripts with an N50 length of 1,041 bp, and 49.9% GC content from eighteen RNA-Seq libraries. These transcripts translated to 437,825 protein coding genes (Table 1). Gene Ontology (GO) annotated DEGs across leaf stages and cell types accounted for 4,339 of the 106,222 (4.08%) *A. madagascariensis* GO annotated genes across all leaf stages and cell samples with a 1% adjusted *P*-value cut-off (Fig. 2A, B). Of the 10,416 DEGs, 2808, 313, 1541, and 1267 genes were upregulated exclusively in pre-perforation, window, mature, and imperforate leaves respectively (Fig. 2A). Between cell types, 482 and 166 genes were exclusively upregulated (at least twofold) in PCD and NPCD cells, respectively. Remaining DEGs were expressed mutually between different combinations of leaf stages (Fig. 2).

**Table 1:** Number of differentially expressed and GO annotated genes across leaf and cell-type samples

Trinity assembly Data	Total
Number of de novo assembled transcripts	908,119
N50 (bp)	1,041
Median transcript length (bp)	374
Average transcript length (bp)	671.74
Percent GC (%)	49.89
Number of protein coding genes	437,825
Number of GO annotated genes	106,222
Number of DEGs	10,416
Number of GO annotated DEGs	4,339

#### Transcriptomic profiles of the lace plant developmental leaf stages

BLAST-based comparisons of assembled transcripts yielded 106,222 GO annotated genes that were homologous to sequences in public databases. Of the annotated genes, 30,932 (29.12%) were most similar to *Arabidopsis thaliana*, 6,509 (6.12%) to *Oryza sativa* and 692 (0.65%) to *Zea mays*.

**Fig. 3:**
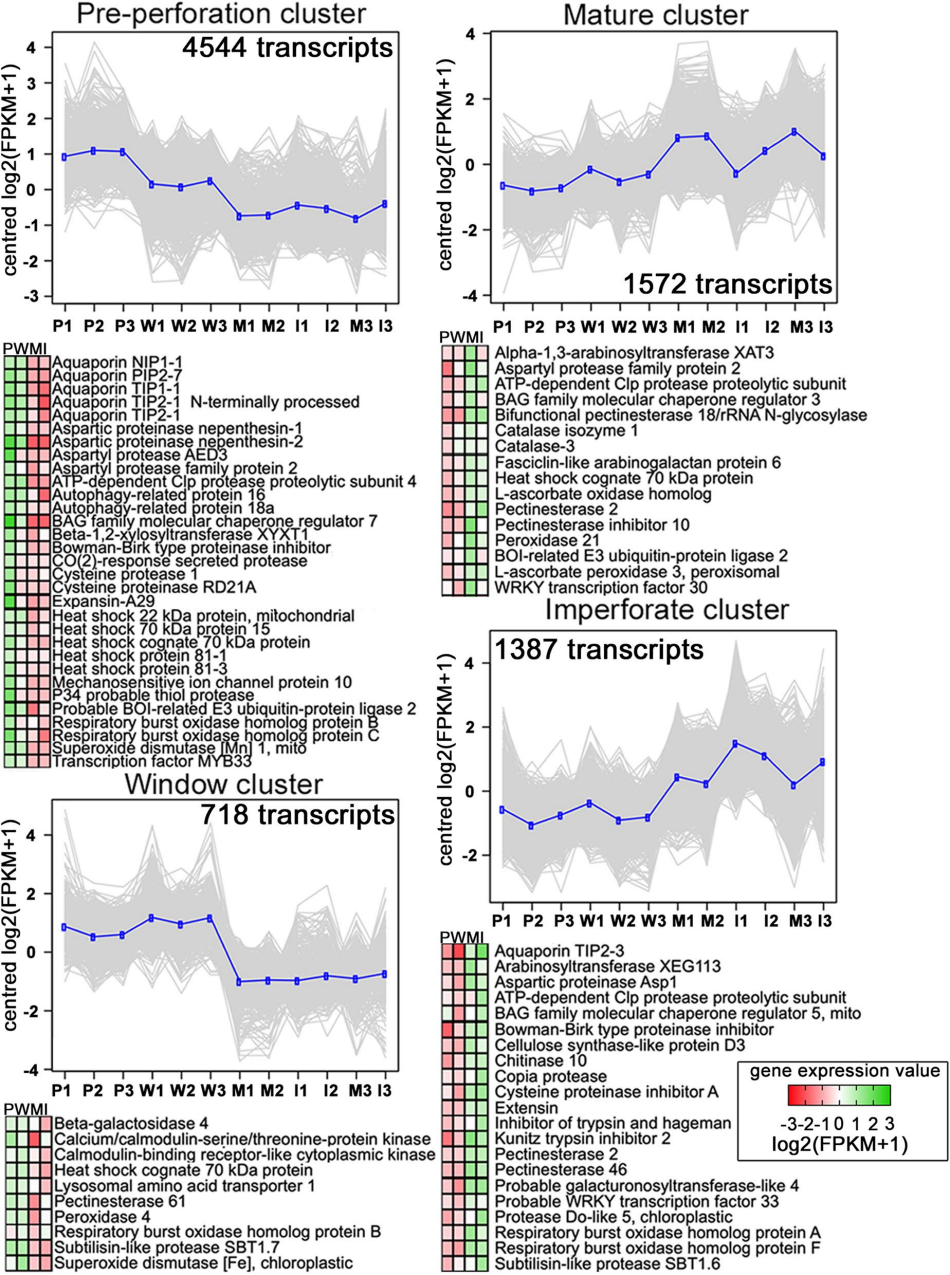
Transcriptomic analysis of lace plant leaf developmental stages. Top: Four main clusters grouped by highest expression in respective pre-perforation (P), window (W), mature (M) and imperforate (I) leaf stage biological replicates (*n* = 3). For each cluster, individual DEG expression values (shown as the transformed log_2_(FPKM + 1) values) are plotted as grey lines and the mean expression profile is highlighted in blue. The total number of DEGs per cluster is shown below each plot (*P* < 0.01, fold change > 2.0). Bottom: Heatmaps of composite gene expression for indicated proteins, with green and red corresponding to high and low gene expression, respectively. Supporting data are found in Additional file 3

*A. madagascariensis* leaf DEGs were divided into four main clusters generated through the tree cutting method of expression patterns across four leaf developmental leaf stages (Fig. 3, Additional file 3). RNA-Seq data were divided into the pre-perforation cluster (4544 DEGs), the window cluster (718), the mature cluster (1572), and the imperforate cluster (1387) (Fold change > 2.0, *P* < 0.01, false discovery rate (FDR) = 1%; Additional file 3). GO enrichment analyses identified biological functions enriched in the four main clusters based on expression patterns across the four stages of leaf development (FDR = 1%, Additional file 3).

The pre-perforation cluster, representing genes that were most highly expressed in the pre-perforation stage leaves and exhibited reduced expression in subsequent stages, included genes encoding for proteins involved in flavonoid biosynthesis, anthocyanin biosynthesis, ethylene-activated signalling pathway, endopeptidase activity, autophagosome formation, and regulation of PCD. The window stage leaf cluster represented genes that were most highly expressed in the pre-perforation and window stages and then reduced in subsequent stages. This cluster included genes encoding for proteins involved in ATP-binding, ATPase activity, ion binding, response to auxin, response to oxygen-containing compounds, peroxidase activity, and hydrolase activity. Both the mature and imperforate clusters represented genes that increased in expression in later leaf development stages, and both contained genes encoding for proteins involved in photosystem I and II, chlorophyll-binding, light-harvesting complex organization, catalytic activity, ion binding, and cell wall biosynthesis.

Taken together, clustering data support the hypothesis that growth and organizational processes are enriched in developing pre-perforation and window stage leaves where metabolic processes must occur to fuel development. Many energy-related metabolic processes occur in the mature and imperforate leaves where development is near completion and flavonoid synthesis is reduced (Fig. 3, Additional file 3). General patterns of gene expression for select biological functions across the leaf clusters showed that genes involved in photosynthesis and negative regulation of PCD are expressed at higher levels in mature and imperforate leaves where PCD is not as active and homeostasis is reached. This suggests that imperforate and mature leaves are the major site of photosynthesis, whereas pre-perforation and window leaves specialize in growth, responding to hormones, and executing PCD. All clusters demonstrated high expression of genes involved in cell wall modifying enzymes such as pectinesterases. However, pre-perforation and window leaves possess a greater number of highly expressed expansins, pectinesterases and subtilisin-like proteases than mature and imperforate leaves. Pre-perforation and window stage leaves represent the culmination of many developmental processes such as regulation of PCD, cell wall organization, lignin, and stomata development. Genes encoding proteins involved in hormone synthesis and transport were found to be differentially expressed between leaf stages. Pre-perforation and window stage leaves had high expression levels of genes encoding for auxin biosynthesis and transport, abscisic acid (ABA) biosynthesis, brassinosteroid (BR) biosynthesis, cytokinin (CK) biosynthesis, gibberellin (GA) biosynthesis, ethylene biosynthesis, ethylene receptor activity, ethylene signalling pathway, jasmonate biosynthesis and salicylic acid (SA) response. Mature and imperforate leaves expressed similar levels of ABA transport genes in comparison to pre-perforation and window stage leaves. In total, early developing leaves revealed an expression pattern of leaf development similar to other monocots like *Agave deserti* and *Z. mays* [21] where expression of most transcription factors (TFs) and hormone biosynthesis tend to be at their highest. Likewise, mature leaves express genes related to photosynthesis [22].

#### Insights from comparative transcriptomics of NPCD and PCD cells

**Fig. 4:**
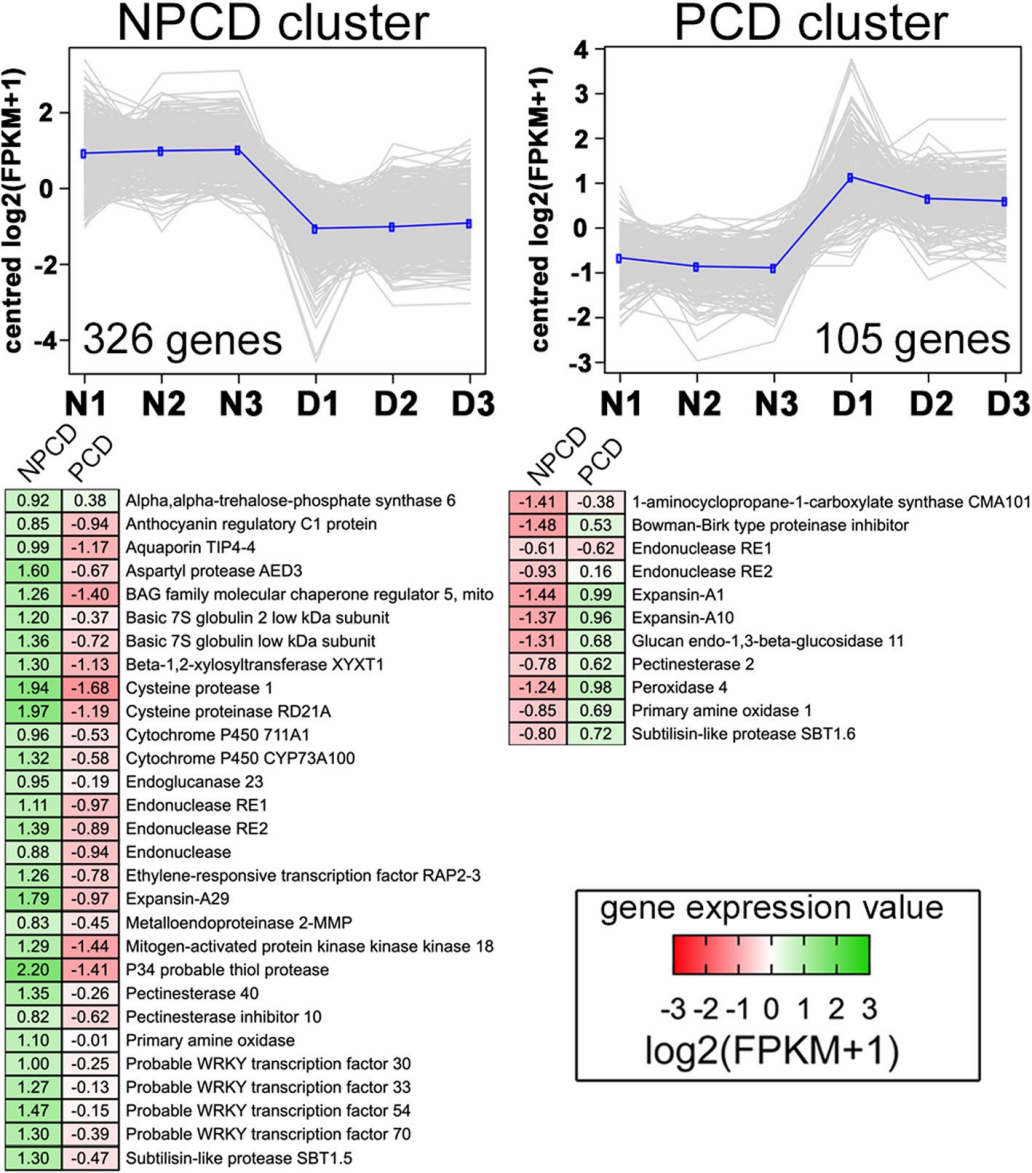
Transcriptomic analysis of NPCD vs PCD cells of the lace plant. Top: Two main clusters grouped by highest expression in NPCD and PCD cell biological replicates (*n* = 3). For each cluster, individual DEG expression values (shown as the transformed log_2_(FPKM + 1) values) are plotted as grey lines and the mean expression profile is highlighted in blue. The total number of DEGs per cluster is shown below each plot (*P* < 0.01, fold change > 2.0). Bottom: Heatmaps of composite gene expression for indicated proteins, with green and red corresponding to high and low gene expression, respectively. Supporting data are found in Additional file5

Laser capture microdissection was used to separate NPCD and PCD cells from *A. madagascariensis* window stage leaves to reveal potential regulators of PCD by RNA-Seq analysis. NPCD and PCD transcriptomes were divided into two main clusters based on cluster analysis expression patterns (Fig. 4) using the tree cutting method. This cluster analysis identified approximately 431 genes that were differentially expressed in either NPCD (326 DEGs) or PCD (105 DEGs) samples with a minimum of a twofold change (*P* < 0.01). In comparison to upregulated genes found in leaf stages; this represents a small fraction of the transcriptome which may be a result of limited biological replicates used in this experiment. We tested for GO enrichment (FDR = 1%; Additional file 5) in each of the NPCD and PCD clusters. The NPCD cluster contained genes encoding for proteins involved in respiratory burst activity, leaf senescence (including negative regulation of senescence), protein autoubiquitination, and the ethylene-activated signalling pathway. The PCD cluster contained genes encoding for proteins involved in ethylene biosynthesis, cell wall modifiers, protease inhibitors, and ROS generation, and PCD regulation (Fig. 4).

**Fig. 5:**
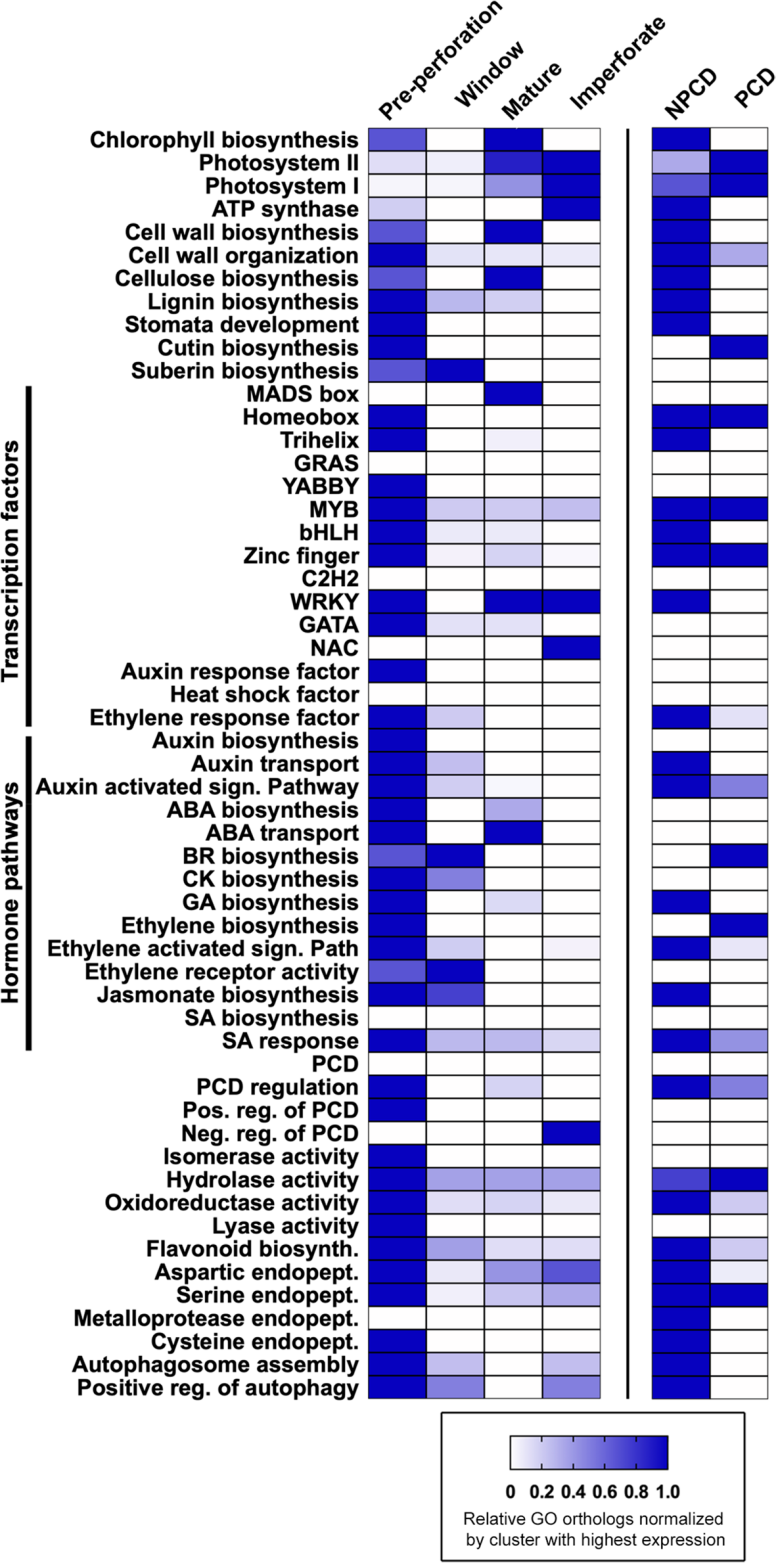
Heat map of GO category orthologs differentially expressed in leaf stages, NPCD and PCD cells. Heatmaps show composite select GO term counts normalized by the cluster with highest count for specific biological processes. The number of GO category orthologs were compared among lace plant leaf stages (left) and between NPCD and PCD cells (right). Colour gradient ranges from white (zero genes upregulated) to dark blue (highest number of genes upregulated). Supporting data are found in Additional file 3 and 5

Comparing the relative GO counts between NPCD and PCD clusters (Fig. 5) we found that NPCD cells upregulated more genes encoding for proteins involved in flavonoid biosynthesis, cysteine protease activity, metalloprotease activity, positive regulation of autophagy, PCD regulation, and cell wall organization. Conversely, PCD cells upregulated more genes involved in ethylene biosynthesis, photosystem II and I, BR biosynthesis, and cutin biosynthesis. NPCD and PCD cells expressed similar numbers of genes in GO categories for homeobox, myeloblastosis (MYB), zinc finger TF families and serine endopeptidase activity, suggesting that these families act as regulatory elements across both cell types during differentiation.

Additionally, five DEGs were selected for fold-change expression validation by quantitative reverse transcriptase polymerase chain reaction (qRT-PCR) across leaf stages and cell types (Additional file 4). The transcripts for *Bag5*, *expansin A-29*, *aquaporin 4–4*, *anthocyanin regulatory protein C1*, *nuclear transcription factor YC-1*, and *α-tubulin* were chosen as validation targets. The fold-change expression of the selected genes normalized by *α-tubulin* followed a similar pattern to that seen in the cluster analysis among different stages of leaf development and between cell types (Additional file 4) and were deemed valid.

### Transcription factors

Most of the DEGs that encode for TFs were found in early developing leaves (Fig. 5, Additional file 3). TFs identified in the pre-perforation leaf cluster included YABBY, MYB, bHLH (basic Helix-Loop-Helix), Zn finger, GATA, C2C2 CO (constans), homeobox, DOF (DNA-binding with one finger), TCP (teosinte, branched1, cycloidea and PCF), and trihelix families (Fig. 5, Additional file 3). These identified families presumably underpin the expression of a broad array of genes important for early leaf development, in particular, the establishment of axial polarity, stomata development, and vascular tissue, as seen *in O. sativa* and *A. thaliana* [23–31]. Mature and imperforate leaves expressed a greater number of genes than pre-perforation and window leaves that encode for MADS-box and NAC (no apical meristem) family TFs which may underpin the promotion of photosynthetic development, lignin, wax and secondary cell wall development which is enhanced in these later developing leaves.

DEGs encoding for TFs were also detected in NPCD and PCD cell DEG expression profiles (Fig. 5, Additional file 5). Trihelix and WRKY families were expressed at a high level in NPCD cells. These identified TFs were most likely responsible for transcribing genes involved in stress response and are known to be upregulated in mesophyll cells of developing *O. sativa* leaves [21]. Two constan family TFs were highly expressed in PCD cells. Constan TF families may be involved in mediating PCD cell collapse or suppressing anti-PCD genes. Both NPCD and PCD cells upregulated an equal number of MYB, Zn finger, and homeobox family TFs. There may be MYB TFs in both types of cells but these two groups possess opposite responsibilities in terms of promoting and suppressing flavonoid biosynthesis. The differences in transcriptional regulation of gene expression between perforating and non-perforating lace plant leaves may control the key programming events that result in differential growth.

### Plant hormones

We detected higher expression patterns for auxin, ABA, CK, GA, ethylene, and jasmonate hormone biosynthesis genes in the pre-perforation cluster (Fig. 5, Additional file 3). The window stage cluster contained the highest levels of expression for BR biosynthesis and ethylene receptor activity. The mature and pre-perforation stage clusters contained the highest levels of expression for ABA hormone transport (Fig. 5, Additional file 3). These results support previous findings of several plant-specific hormones being involved in PCD signalling, including SA, jasmonic acid, ABA, GA, and ethylene [32–34]. The hormone biosynthesis patterns observed in early lace plant development are similar to the monocot leaves of *A. deserti, Agave tequilana* and *Z. mays* [21, 22].

To date, several pharmacological whole plant experiments have revealed how lace plant perforation formation is dependent on auxin, and ethylene biosynthesis [35–37]. Further work is required to disentangle the roles of each plant hormone in mediating lace plant leaf development from perforation formation, outside of ethylene which has been studied extensively.

Expression patterns in the NPCD cell cluster included more highly expressed genes related to auxin transport, auxin signalling pathway, GA biosynthesis, ethylene activated signalling pathway, jasmonate biosynthesis and SA response in comparison to the PCD cell cluster (Fig. 5, Additional file 5). PCD cells upregulated 1 gene encoding for ethylene biosynthesis relative to NPCD cells. Ethylene biosynthesis and ethylene receptors are involved in promoting PCD in cells destined to die in the areoles of lace plant leaves [35, 37]. Additionally, PCD cells upregulate more genes associated with BR biosynthesis in comparison to NPCD cells. BRs are believed to mediate the timing of ROS production, and in turn, PCD execution in tapetal cells of *Solanum lycopersicum* [38]. BRs may also play a similar role in PCD cell triggering, as suggested by the higher expression of genes involved with BR synthesis. NPCD cells upregulated ethylene response factor (ERF) RAP2-3 in comparison to PCD cells. ERF-RAP2-3 has been identified as playing an important role in ethylene mediated hypoxia stress in *A. thaliana* seedlings [39] and may play a role in protecting NPCD cells from PCD cells which accumulate superoxide during PCD execution. Together, our results support the hypothesis that plant hormones are involved in PCD, and targeted approaches are needed to disentangle their specific roles and functions.

### Anthocyanin biosynthesis enzymes

**Fig. 6:**
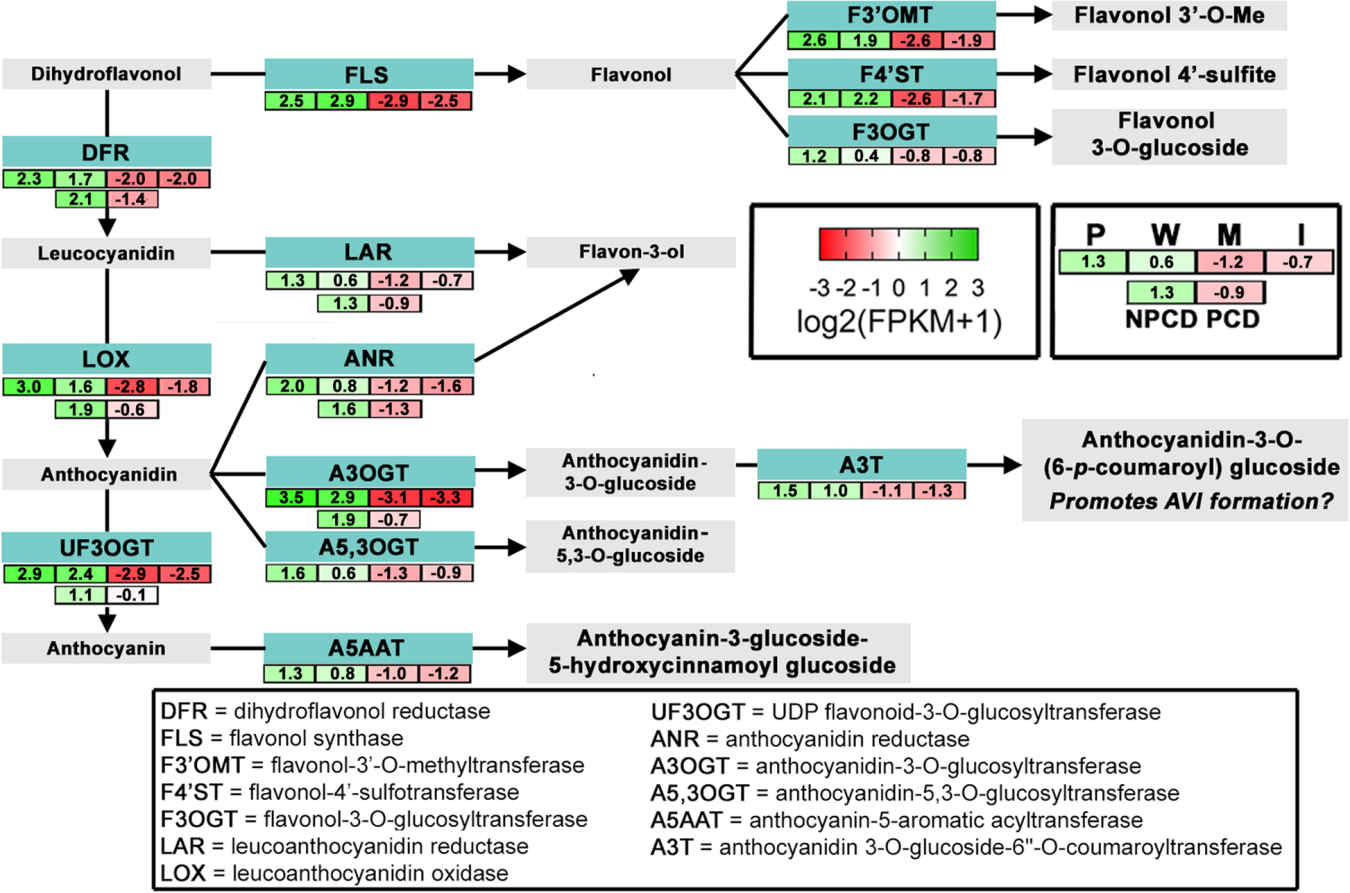
DEGs involved in flavonoid biosynthesis. General late-flavonoid biosynthetic pathway (grey boxes) and genes (cyan boxes) expression in pre-perforation (P), window (W), mature (M) and imperforate (I) leaf stages (4-box expression comparison strings) or NPCD and PCD cell samples (2-box expression comparison strings). Detailed gene expression data provided in Additional file 3 and 5. Expression levels displayed in log_2_(FPKM + 1) across samples. Green indicates high expression values; red indicates low expression values. Pathway modified from Argout et al., (2008) [41]

Forty-six upregulated genes were categorized as enzymes with flavonoid biosynthesis, isomerase, hydrolase, oxidoreductase or lyase activities in the pre-perforation leaf cluster while < 10 genes for these enzymes were found in each of the window, mature, and imperforate leaf clusters (Fig. 5, Additional file 3). Eight of the upregulated genes in the pre-perforation cluster encoded for the flavonoid biosynthesis pathway. Pre-perforation and window leaves upregulated genes that promote the biosynthesis of early stage and late-stage flavonoids as well as downstream transferase enzymes for promoting the synthesis of anthocyanins, flavonols and anthocyanidins. NPCD cells also upregulated 5 genes that encoded for flavonoid biosynthesis in comparison to PCD cells (Fig. 5, Additional file 5). Of the genes that are involved in flavonoid biosynthesis only *flavonone 3-dioxygenase 2,* which produces dihydroflavonol [40], was upregulated in mature leaves and imperforate leaves as well as PCD cells (Additional files 3 and 5). Upregulated genes that encode for enzymes that promote anthocyanin biosynthesis are summarized in Fig. 6.

The most notable feature of lace plant leaf development is the visible gradient of anthocyanin pigmentation during PCD in early developing leaves. Previous studies have investigated the role of exogenous ROS and antioxidants on lace plant leaf development, and found that they are key regulators of the establishment of perforation formation in the lace plant model system [13]. Pre-perforation and window stage leaves have the highest levels of anthocyanin compared to imperforate and mature leaves [13, 42]. Our results are consistent with these findings, as pre-perforation leaves, window leaves, and NCPD cells expressed genes encoding enzymes that promote the biosynthesis of glucoside constituted anthocyanins (Fig. 6). The upregulation of anthocyanidin-3-O-glucosyltransferases (A3OGT), anthocyanidin-5,3-O-glucosyltransferases (A5,3OGT), and anthocyanin-5-aromatic acyltransferase (A5AAT) suggests that anthocyanidin-3-O-glucosides, anthocyanidin-5,3-O-glucosides, and anthocyanidin-3-glucoside-6-hydroxycinnamoyl glucosides are being actively synthesized in early-stage leaves, leading to the accumulation of anthocyanins. The presence of pink-red coloured anthocyanin vacuoles in window stage leaves is consistent with the hypothesis that the vacuole has a pH of ~ 5.5 pH which favours the formation of flavylium cations [43].

Recent attention has been drawn to the formation of anthocyanin vacuolar inclusions (AVIs) in window stage leaves, which then dissipate as leaves enter maturity (Gunawardena lab, 2020 unpublished data). Kallam et al. (2017) [43] recently reported that the composition of anthocyanin substituents or decorations determined the solubility of AVIs in tobacco lines. The aromatic acylation of anthocyanidin 3-O-glucoside by anthocyanidin 3-O-glucoside-6″-O-coumaroyltransferase (A3T) promotes the formation of AVIs, while the rhamnosyl decorations decrease the formation of AVIs by competition. The absence of upregulated aromatic rhamnosyltransferases or malonyltransferase DEGs (which inhibit AVI formation) in early leaves suggests a role for AVIs in mediating proper leaf development [43]. NPCD cells upregulated genes encoding for anthocyanidin 3-O-glucosyltransferases, consistent with the promotion of ROS scavengers and protection from PCD. The identification of anthocyanin compounds across developmental leaf stages and NPCD cells is currently under investigation using global mass spectrometry.

### Potential regulators of programmed cell death

#### Cell wall modification enzymes and aquaporins

Lace plant perforation formation relies on thin cuticle erosion, removal of polysaccharides and degradation of cellulose [44] to mediate perforation expansion. The deposition of suberin is imperative to prevent infection and loss of nutrients in NPCD cells while neighbouring PCD cells collapse. We found that the window stage cluster had high expression of the greatest number of suberin biosynthesis-related genes (Fig. 5, Additional file 3), which is consistent with the hypothesis that suberin deposition is most active during the window stage of development [12].

Pre-perforation and window stage clusters contain high expression of 67 and 7 genes, respectively, that were categorized under either cell wall organization or cell wall biosynthesis. Mature and imperforate clusters showed high expression of 9 and 5 genes respectively. The NPCD cluster showed high expression of 4 genes, while the PCD cluster showed high expression of 1 gene (Fig. 5, Additional file 5). All clusters contained higher expression for orthologs of hydrolases, pectinesterases, glucosidases, and xyloglucan glycosyltransferase activities. Mature and imperforate leaves upregulated no genes that encode for expansin enzymes.

The facilitation of cell expansion and cell wall loosening is important for not only the growth of developing lace plant leaves but also the execution of PCD cells and reorganization of NPCD cells. Pre-perforation and window stage leaves upregulated several genes that encode for xyloglucan endotransglucosyltransferases, expansins (*expansin-A29* detected by qRT-PCR, Additional file 4), and pectinesterases (Fig. 3) which are responsible for loosening and reorganizing the cell wall during growth [45–47]. Mature and imperforate stage leaves followed a similar pattern but did not upregulate genes for expansins (Fig. 3). This likely indicates that this activity is no longer needed once leaf maturity is reached.

Aquaporins play an important role in cell expansion by controlling water uptake [48]. Pre-perforation and window stage leaves upregulated several genes encoding for tonoplast intrinsic protein (TIP), nodule intrinsic protein (NIP), and plasma membrane intrinsic protein (PIP) aquaporins. NPCD cell upregulated a TIP4-4 aquaporin gene (Fig. 4). *TIP4-4* was detected by qRT-PCR (Additional file 4). PCD cells upregulated a TIP2-3 gene and mature and imperforate leaves upregulated both. The expression of aquaporin genes in all samples suggests that cell expansion by vacuole enlargement is needed throughout leaf development, and as well as in differentiating NPCD and PCD cells to control the progression of expansion or cell burst [48].

#### Heat shock proteins

Heat shock proteins (Hsps) are synthesized in response to stress to maintain homeostasis by refolding proteins before they become irreversibly denatured [49, 50]. The pre-perforation leaf cluster contained 25 genes and the window leaf cluster contained 2 genes categorized as protein folding. Mature and imperforate leaf clusters contained 1 and 2 genes under unfolded protein folding, respectively (Fig. 3, Additional file 3). The NPCD cell cluster contained 1 gene and the PCD cell cluster contained 0 genes categorized under unfolded protein folding (Additional file 5). ATP-dependent molecular chaperones such as Hsp81-1, Hsp81-3, and Hsp70-15 were upregulated in pre-perforation and window stage leaves.

In *O. sativa* protoplast models, mtHsp70 overexpression prevents ROS burst and PCD in protoplasts under oxidative stress [51]. In contrast to suppressing stress induced PCD, Hsp70 of *Capsicum annuum* promotes the hypersensitive response in infected *Nicotiana benthamiana* leaves [52] by nuclear localization of an effector protein. Hsp70s and their respective Bcl-2-associated athanogene (Bag) proteins can modulate animal PCD and many cellular processes [1], warranting their investigation in lace plant PCD. Experimental treatment with the Hsp70 inhibitor chlorophenylethynylsulfonamide (PES-Cl) caused a significant decline in the number of perforations, caspase-like activity and anthocyanin levels in window stage leaves [42], suggesting Hsp70 plays a role in mediating lace plant PCD. Hsp70 proteins are developmentally regulated and significantly higher in pre-perforation and window stage leaves, which is consistent with the expression pattern of an Hsp70-15 gene (Fig. 3). While our results support the hypothesis that Hsp70 activity affects lace plant leaf development at a threshold level, where it localizes is still unknown.

Genes for lace plant homologs of *O. sativa* Hsp81-1 and Hsp81-3 were transcriptionally upregulated in pre-perforation stage leaves (Fig. 3, Additional file 3). Hsp81s promote salt stress tolerance in *O. sativa* and over-expression experiments in *A. thaliana* show that it promotes heat tolerance [53, 54]. Proteomic models predict that AtHsp81 can form a complex with AtHsp70 [55] most likely for protein quality control, and our results suggest that both lace plant homologs of Hsp70 [42] and Hsp81 are being synthesized for maintaining protein homeostasis during early leaf development. Additional pharmacological whole plant experiments are required to improve characterization of Hsps and Bag protein function in lace plant development.

#### Bag proteins

The Bag protein family has gained recent attention in the field of plant developmental biology for their role in mediating PCD, senescence, and autophagy in several plant systems [1, 55]. Bag proteins are major nucleotide exchange factors for Hsp70 [56] and help accelerate the Hsp70 protein fold cycle and modulate PCD pathways in animals and plants [57, 58]. The Hsp70 co-chaperone Bag7 was more highly expressed in pre-perforation and window leaves. Bag3 and mitochondrial (mt) Bag5 were upregulated in mature and imperforate leaves (*mtBag5* detected by qRT-PCR, Additional file 4). Expression values for mtBag5 were significantly higher in NPCD cells in comparison to PCD cells (Fig. 4, Additional file 5).

In the lace plant, we found that pre-perforation and window stage leaves upregulated genes encoding homologs of *A. thaliana* for Bag7 (Fig. 3, Additional file 3). Bag7 is ER-localized in *A. thaliana* and is involved in the unfolded protein response, and can localize to the nucleus to interact with multiple proteins to modulate PCD pathways [59, 60].

Mature and imperforate lace plant leaves upregulated Bag3 and Bag5 homologs relative to pre-perforation and window leaves (Fig. 3), whereas NPCD cells upregulated a Bag5 homolog compared to PCD cells (Fig. 4). The role of Bag3 involvement in human cargo mediated autophagy has made it a potential cancer therapy target, but its role in plant PCD is unknown [61]. In *A. thaliana* leaf systems, Bag5 has been found to bind heat shock cognate 70 (Hsc70) and localize to the mitochondria to promote ROS generation and clearing of chlorophyll while leaves are under senesence [58]. The fact that Bag5 genes were upregulated in NPCD cells provides an opportunity to investigate the possible role of this gene in separating NPCD cells from PCD cells or preventing mitochondrial burst [62]. Plant Bag5 plays a role in regulating Ca^2+^ in the mitochondria and possibly the outcomes of mitochondrial stress response in NPCD cells. Bag family proteins seem to play an important regulator role in plant PCD but their precise biochemical roles are still unknown in plants [61].

#### Autophagy-related proteins

Dauphinee et al. (2019) found that autophagy predominately contributes to cell survival and that there is no clear evidence for the direct involvement of autophagy and the induction of PCD during the perforation formation of lace plant leaves [14]. Only pre-perforation and window stage leaves were found to upregulate genes in the GO category of autophagosome assembly such as homologs of AuTophaGy-related protein 16 (Atg16) and Atg18a (Fig. 3 and 5, Additional file 3). The pre-perforation cluster contained a SNF1-related protein kinase catalytic subunit alpha KIN10 and a lysosomal amino acid transporter 1, both of which are involved in autophagy regulation. Mature leaves, imperforate leaves, and NPCD cells (in comparison to PCD cells, Fig. 3 and 4) upregulated WRKY33, a TF involved in the positive regulation of autophagy [63]. WRKY33 was upregulated in NPCD cells relative to PCD cells, suggesting a requirement for stress resistance in NPCD cells during perforation development. WRKY33 is important for plant resistance to necrotrophic pathogens and interacts with the Atg18a in the nucleus [64] in response to biotic stress.

We detected higher expression of genes for Atg18a and Atg16 in pre-perforation and window stage leaves, suggesting changes in the regulation of autophagosome formation and mitophagy occur during plant developmental PCD. There are eight members in the *AtATG18* gene family (*AtATG18a*–*AtATG18h*) [63]; each member has a different expression pattern, and only *AtATG18a* shows an increased transcript level under starvation conditions and during senescence in *A. thaliana*. *AtATG18a* expression is also upregulated and is required for autophagy during oxidative, salt, and osmotic stresses. RNA interference (RNAi) of *AtATG18a* makes plants autophagy-defective and more sensitive to various stress conditions [63, 65–67]. Atg16 oligomerizes to form an Atg12-Atg5·Atg16 complex that is essential for autophagy [68].

The *A. thaliana* protein kinase SNRK KIN10 has been shown to induce several autophagy genes such as Atg8, a protein found to be developmentally regulated in lace plant leaves [69, 70]. During early leaf development, the level of expression of genes involved in photosynthesis is low in comparison to mature and imperforate leaves. Prolonged stress often causes decreased photosynthesis and increased ROS accumulation, which can trigger PCD pathways [71]. Autophagy is used in early leaf development to provide energy while photosynthesis and chlorophyll genes are not upregulated until maturity and optimal photosynthesis activity is reached. It is likely WRKY33 is transcribed to increase autophagosomes formation during early leaf development and to promote survival in stressed NPCD cells.

### Regulators of programmed cell death

Genes falling under the GO category “PCD regulation” were differentially expressed across lace plant leaf stages and between NPCD and PCD cells. We found that the pre-perforation leaf cluster contained homologs for L-type lectin domain kinase IX.I and mechanosensitive ion channel protein 10 (MSL10). MSL10 promotes PCD in response to pathogen invasion, and mechanical stress in *A. thaliana* [72–74]. Additionally, the pre-perforation cluster contained genes encoding for a BOI-related E3-ubiquitin ligase 2. BOI E3-ubiquitin ligases are capable of inhibiting PCD by limiting α-picolinic acid generation and by ubiquitination of apoptotic inhibitors [75, 76]. The upregulation of MYB33 in pre-perforation and window stage leaves (Fig. 3, Additional files 3) is of particular interest for further lace plant investigations, due to its role in the promotion of PCD in anthers and seeds of other monocots like *Hordeum vulgare* and *O. sativa* [77].

The mature and imperforate leaf stage clusters contained genes encoding a BOI related E3 ubiquitin ligase 2 and a respiratory burst oxidase homolog-F (Rboh-F) respectively. BOI related E3 ubiquitin ligase 2 genes negatively regulate PCD by suppressing ROS generation [75, 76] which is consistent with mature and imperforate leaves where perforation formation and superoxide accumulation is less active [13, 42]. Rboh-F is mostly responsible for ROS generation by ABA signaling in *A. thaliana* systems [78] and is implicated in immunity.

We also observed differential expression patterns for PCD regulation genes across NPCD and PCD cells (Fig. 5, Additional file 5). NPCD upregulated genes encoding for aspartyl protease AED3 and ERF-RAP2-3 which have been previously described as pro-PCD contributors. PCD cells upregulated a gene encoding for primary amine oxidase 1 (PAO1) relative to NPCD cells. PAOs have been shown to play a role in generating ROS in differentiating tissue during organ development and during PCD [79, 80]. Pro-PCD genes such as aspartyl protease AED3 upregulated in NPCD was an unexpected result and may potentially be explained by these genes having a function to promote a stress response or senescence in NPCD cells during PCD activation.

#### Plant proteases and programmed cell death

The pre-perforation, window, mature and imperforate clusters were all found to contain upregulated genes encoding for enzymes with endopeptidase activity. The pre-perforation leaf cluster shows 59 genes encoding for enzymes with aspartic, serine, and cysteine endopeptidase activity (Fig. 5, Additional file 3). NPCD cells upregulated 27 genes encoding for enzymes with aspartic endopeptidase activity (2 for PCD cells), 1 gene with serine activity for both cell types, 4 with cysteine activity (versus for 0 in PCD cells), and 1 with metalloprotease (0 for PCD; Fig. 4 and 5, Additional file 5).

Previous research has pinpointed the roles of caspase-like enzymes in plant development as PCD initiators or executors [81–83]. Subtilisin-like proteases have potential PCD regulation roles with an autocatalysis activity-containing domain that re-enters the cell once the prodomain is removed to execute PCD, and all lace plant leaf stage clusters upregulated several subtilisin-like proteases. The similar expression of *subtilisin-like proteases* across leaf stages is consistent with a role in leaf remodelling and homeostasis. All leaf stages and NPCD cells upregulated a Bowman-birk serine protease inhibitor. Serine protease inhibition activity may indicate a role for coordinated inhibition of serine endopeptidase activity for proper PCD execution.

Multiple cysteine proteases have documented roles in developmental plant PCD. For example, cysteine protease 1 mediates tapetal PCD in *A. thaliana* [82] and cysteine protease R2D1A is found to enhance PCD in innate immunity of *A. thaliana* [83, 84]. Cysteine protease activity can be tightly controlled by the activity of serpin and Kunitz protease inhibitors (reversible inhibition [83]), which are upregulated in mature and imperforate leaves in the form of Kunitz protease inhibitor 2 and cysteine protease inhibitor A. Protease cascades may trigger lace plant leaf PCD. Inhibitors such as the Kunitz and cysteine inhibitors in mature leaves could play a role in stopping the effector phase of cysteine protease activity and preventing PCD from becoming active again [85]. We found that both genes for cysteine protease 1 and RD21A previously mentioned are transcriptionally upregulated in NPCD cells. The reason(s) for the accumulation of mRNA for these proteases in cells destined to survive is still uncertain, this could suggest they play an important role in mediating PCD cell collapse.

Aspartyl proteases also regulate plant developmental PCD; specifically, the deletion of reproductive tissues [86, 87]. In lace plants, pre-perforation, window stage leaves, and NPCD cells all had high expression of genes encoding for aspartyl protease AED3, which is involved in PCD (Fig. 3 and 4; Additional file 3 and 5). Aspartyl protease AED3 may be upregulated for transdifferentiating NPCD cells into endodermis during PCD progression.

A metalloproteinase 2-MMP gene was upregulated in NPCD cells in comparison to PCD cells (Fig. 4). SI2-MMPs have been found to inhibit epidermal cell death in *S. lycopersicum L* and, in contrast, 2-MMP promotes early senescence in *A. thaliana* [88, 89]. The upregulation of *2-MMP* in NPCD cells may indicate their role in differentiating NPCD from PCD cells, or inhibiting PCD execution.

The protease substrate phosphoenolpyruvate carboxykinase 1 (PEPCK1) can be cleaved by *A. thaliana* metacaspase 9 (MC9) and MC9 in turn promotes the clearance of root xylem tissue [90] and possibly gluconeogenesis [91]. A gene encoding an *A. thaliana* PEPCK1 homolog [85] was upregulated in our imperforate leaf cluster (Fig. 3, Additional file 3). The cleavage of PEPCK1 by MC9 may promote gluconeogenesis in imperforate leaves and supports the hypothesis that imperforate leaves serve to generate energy for the lace plant before undergoing rapid senescence.

Interestingly, our RNA-Seq analysis did not reveal differential expression across leaf stages or cell types of any lace plant genes encoding for vacuolar processing enzymes (VPEs), which are known to play a major role in developmental PCD of lace plant leaves [35, 92]. Using qRT-PCR methods, Rantong & Gunawardena (2018) showed that transcript levels of *Am*VPE-1 and *-*2 are significantly upregulated in early developing leaves and window leaves (normalized to *Am*Actin). Previous work has highlighted the fact that VPE activity is important for vacuolar collapse in lace PCD cells. The absence of this process from results of our study and that of Rantong & Gunawardena (2018) suggests that autoprocessing and post-translational modification of the VPE protein might explain its functional activity, rather than just higher accumulation of mRNA [92].

## Conclusions

**Fig. 7:**
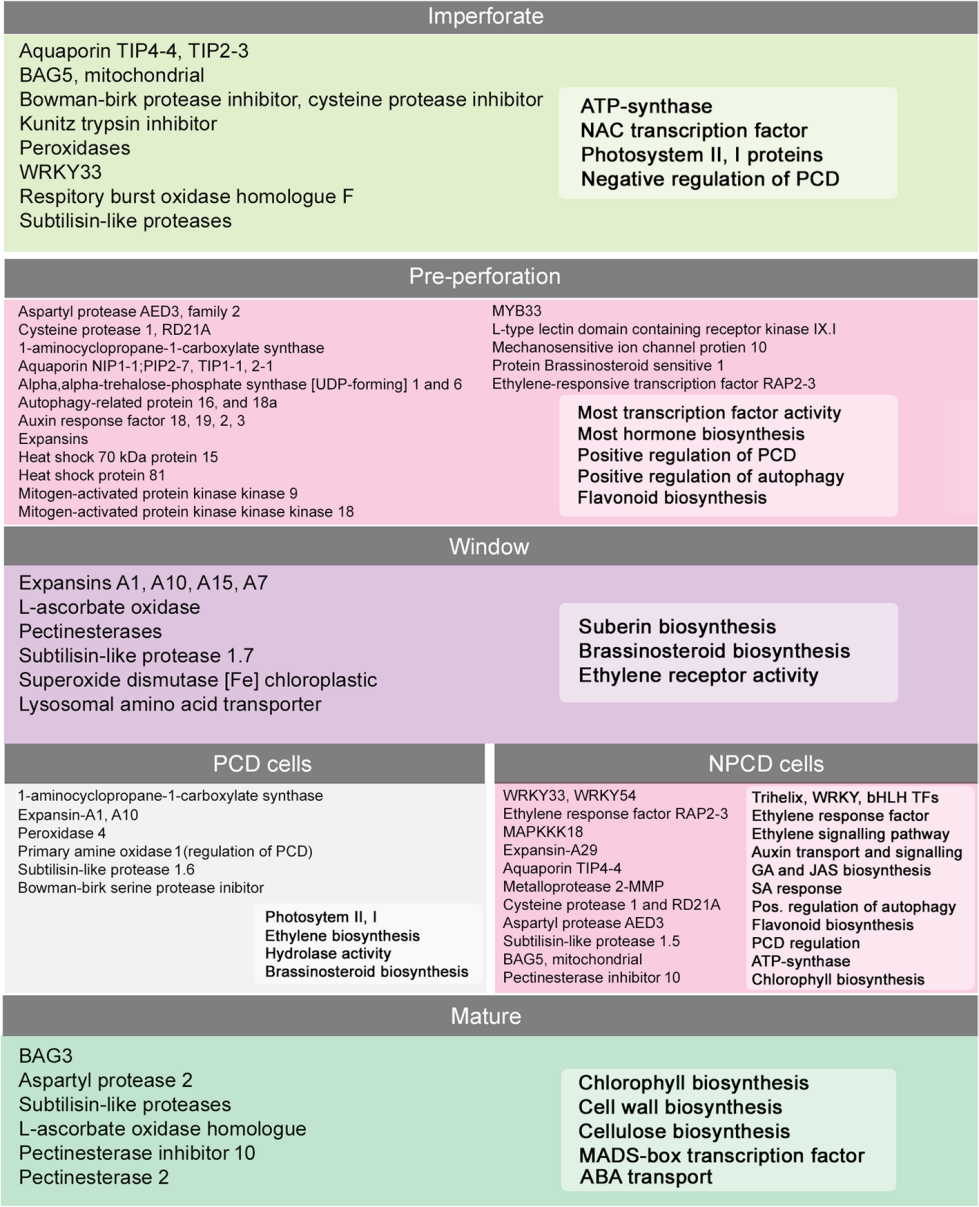
Summary of differentially expressed genes involved in lace plant leaf remodelling, based on RNA-Seq data. Summary of transcriptionally upregulated genes involved in lace plant leaf remodelling and differentiation of NPCD and PCD cells. GO terms bounded in white shaded boxes are most highly expressed GO counts in respective sample cluster (Additional files 3 and 5)

The cellular dynamics and chronological events of lace plant leaf PCD are well documented. Here, we investigate the molecular basis of this process by characterizing the transcriptomic profiles of different stages of leaf development and PCD and NPCD cells isolated from window stage leaves. We profiled DEGs to summarize genes controlling the mechanism of developmental PCD and leaf remodelling (Fig. 7).

Based on comparative transcriptomics our results support the hypothesis that NPCD and PCD cell differentiation is mediated by a differential balance of plant hormones and TF activities that both promote and limit the PCD pathway. GO enrichment analyses of DEGs suggest that autophagy, cell expansion, protease activity, ROS generation, and flavonoid biosynthesis work in concert to ensure promotion of perforation expansion during lace plant leaf development. The high level of expression of genes involved in these diverse biological functions differed significantly between early and late lace plant leaf developmental stages, indicating their involvement in regulating perforation initiation, execution, and leaf growth. The results of our investigation into the lace plant transcriptome and expression patterns reveal a variety of candidate genes with possible involvement in the initiation and progression of lace plant leaf cell death, generating new hypotheses and providing novel insights into plant developmental PCD. Future experiments on candidate DEGs will be required moving forward to characterize and confirm protein functionality in lace plant leaf perforation formation.

All things considered, the *A. madagascariensis* transcriptome data generated and analysed herein exemplifies the power of de novo Illumina-based RNA-Seq. Our transcriptomes serve as both a high-quality gene discovery resource and a framework for the detection of physiological changes through gene expression profiling. Combined with additional transcriptome annotation tools, experimental observations from model plant species will undoubtedly facilitate deeper insights into the biology of PCD and remodelling of lace plant leaves in the future.

## Methods

### Plant tissue culturing

Lace plant cultures were propagated aseptically as described in Gunawardena et al. (2006) [93]. Lace plant corms were cultured in Magenta GA-7 boxes, embedded in 100 mL of solid MS media made of 1.5% plant tissue culture agar (w/v, Phytotechnology Laboratories) in liquid MS [3% sucrose (w/v), 0.01% Myo-inositol (w/v), 0.215% MS basal salts (w/v, Phytotechnology Laboratories), 0.0025% thiamine-HCl (v/v), pH 5.7] and then submerged under 150 ml of liquid MS. Plant cultures were grown at 24 °C and exposed under light levels of 125 μmol m^−2^ s^−1^ on 12 h light/dark cycles with daylight deluxe fluorescent light bulbs (Philips). *A. madagascariensis* (Mirbel) H. Bruggen corms were originally purchased from The PlantGuy (AB, Canada).

Cultures grew for 30 days or until each plant produced 3 perforated mature leaves. One of each imperforate, pre-perforation, window stage leaves, and one of the most recently developed mature leaf were separated from one whole plant culture and washed thoroughly with distilled water before leaf tissue was excised from the midrib and flash frozen for downstream molecular work. Three independent experiments were carried out. For each experiment, one of each leaf stage was taken from one whole plant culture.

### PCD and NPCD cell preparations

A Zeiss PALM Laser Capture Microdissection and Imaging System (North York, ON, Canada) was used to separate PCD and NPCD cells. The cells were collected from the areoles of window stage leaves, where each type is visibly distinguishable by colour. Cell type population samples were collected from 4 separate areoles (approximately 2000 cells per areole) per window stage leaf before being flash-frozen. Three independent experiments per cell type were carried out. For each experiment, one window stage leaf was taken from one whole plant culture. A sample diagram of the laser capture and catapult process of the leaf tissue sample is provided in Additional file 2.

### RNA extraction and quality control

RNA was extracted from leaves of four developmental stages, and from the two different cell types (NPCD and PCD cells). RNA was extracted from 40 mg of flash-frozen, midrib free leaf lamina tissue from one of each imperforate, pre-perforation, window or mature stage leaves from 3 different whole-plant cultures as per instructions for the ReliaPrep RNA Kit (Promega). RNA samples were treated with DNAse I (Thermo Fisher). Eluted RNA quantity was estimated using a Nanodrop spectrophotometer (Thermo Fischer) and a RNA integrity number (RIN) was determined using a Bioanalyzer (Agilent Technologies Inc., Santa Clara, CA, USA). Only RNA samples with a RIN ≥ 6.5 were approved for cDNA conversion.

### cDNA library preparation and Illumina sequencing

cDNA library preparation and sequencing were performed by Génome Québec (Montréal, QC, Canada). Eighteen paired-end RNA-Seq libraries of length 100 bp were generated on an Illumina NovaSeq6000 (CA, USA) using strand-specific Trueseq protocols. The raw read data obtained were deposited to NCBI and are accessible under the SRA. SRA accession IDs: SRR10524134-SR10524151 and BIOPROJECTID: PRJNA591467. Data were first inspected for quality by analysing FastQ files with FastQC [94]. Reads of low quality and containing adapter contaminations were trimmed with Trimmomatic v.0.35 [95] with a k-mer size of 25 and with parameters: ILLUMINACLIP:TruSeq3-PE.fa:2:30:10 SLIDINGWINDOW:5:0 MINLEN:50. The quality of trimmed reads was assessed using FastQC v0.11.2 **(**http://www.bioinformatics.babraham.ac.uk/projects/fastqc/).

### Transcriptome de novo assembly

High-quality adapter free reads were used to construct a de novo assembly with Trinity v2.3.1 [96] with default settings. Quality evaluation of assemblies was considered with major bioinformatics indicators such as transcript mean length, GC%, and an N50 value (Table 1). The Trinity pipeline clusters de novo assembled transcripts into genes and isoforms, and we worked only with ‘genes’ datasets, with the highest expressed isoform chosen as the representative for each gene. This Transcriptome Shotgun Assembly project has been deposited at DDBJ/ENA/GenBank under the accession NO. GJFM00000000. The version described in this paper is the first version, GJFM01000000.

### Transcript quantification and identification of differentially expressed genes

The abundance of each gene was calculated by aligning reads from each sample to our de novo transcriptome with RNA-Seq by Expectation–Maximization (RSEM) [97] for each library. The trimmed mean of *M*-values (TMM) method [98] was used to calculate the normalization factors (one calculation for NPCD vs PCD cells and one calculation for comparisons among the leaf stages). Using Trinity [99], expression normalization was performed using TMM, following fragments per kilobase of exon model per million reads (FPKM) calculations. DEGs among the leaf stage and between the cell type libraries were identified using the Empirical Analysis of Digital Gene Expression (edgeR) statistical package [100] (http://bioconductor.org/packages/release/bioc/html/edgeR.html) performed with R (v3.3.2; R Core Team 2015). Genes that were more than twofold differentially expressed with an FDR of 1% were defined as differentially expressed [101].

### Cluster analysis

Expression patterns of genes among leaf stage samples and between NPCD and PCD cells were separated using cluster analysis of DEGs. Hierarchical clustering of normalized gene expression was achieved using centralized and log_2_(FPKM + 1) transformation [99] and tree cutting at a depth of 40%, with heatmap visualization performed using R and the package Superheat [102].

### Annotation and GO enrichment analysis

To identify the genes and functions associated with each transcript, assembled transcripts were annotated using Trinotate [103] and public genome and functional repositories. We annotated transcripts based on top matching sequence similarity to orthologs in public databases, including GO, the Kyoto Encyclopedia of Genes and Genomes (KEGG), the euKaryotic Ortholog Groups database (KOG), the Swiss Protein Resource (Swiss-Prot), and the Panther Database, using the BLASTX method with a cut-off *E*-value of 10^−5^ [104–106]. To eliminate transcripts derived from foreign organisms and lab contaminants, genes of non-plant origin were removed. Selected annotation data was included with Additional file 3 and 5.

GO-enrichment analysis was carried out using the Plant Transcription Factor Database v5.0 [107] program based on Fisher’s Exact Test with multiple testing correction of FDR = 1%. GO analysis was performed by comparing the GO terms in the test sample to the GO terms in the background reference of the entire lace plant de novo transcriptome generated from all samples.

### Validation with qRT-PCR

Five selected DEGs (*Bag5*, *expansin A-29*, *aquaporin 4–4*, *anthocyanin regulatory protein C1*, *nuclear transcription factor YC-1*, and *α-tubulin*) were used to verify the expression results of RNA-Seq by using the ΔΔC_T_ method. RNA from lace plant leaf stages and isolated cell types were collected and extracted as described above. Single-strand cDNA was synthesized using SuperScript®III First-Strand Synthesis System for qRT-PCR (Invitrogen, Burlington, ON, Canada) and oligo dT_20_ following the manufacturer’s instructions. qRT-PCR was conducted on a Rotor-Gene RG-3000 system (Corbett Research, Sydney, NSW, Australia) using 0.5 μl cDNA as a template and 0.4 mmol l^−1^ primers for all selected genes (Additional file 6) under the following conditions: 5 min at 94 °C, 35 cycles of 30 s at 94 °C, 30 s at 54 °C for all chosen genes and 1 min at 72 °C, followed by 72 °C. qPCR was conducted using a QuantiFast® SYBER® Green PCR Kit (Qiagen, Mississauga, ON, Canada). Melt curve analysis was conducted by Rotor-Gene 6 Software and experiments with at least 90% efficiency were used for analysis (Corbett Research). The experiment was performed in triplicate using three biological replicates of imperforate, pre-perforation, window, and mature stage leaves as well as NPCD cells and PCD cells. cDNA copy numbers for five chosen genes (Additional file 4) were determined from a standard curve of Ct values (R^2^ > 0.99) and normalized against the *α-tubulin* isoform [37].

### Image analysis and processing

Images of leaf layouts were obtained using a Nikon L110 digital camera. Photoshop and Illustrator (Adobe Creative Cloud; Adobe Systems Inc.) were used to prepare images and remove backgrounds of detached leaves for publication. A Nikon AZ100 microscope acquired micrographs of leaf stages. Image adjustments were made evenly within and consistently across figures and included background removal, as well as fine tuning of brightness, contrast and colour balance using Photoshop.

### Statistical analysis and data representation

One-way ANOVA followed by a Tukey test was used to identify significant differences among leaf stage means for qRT-PCR validation experiments, and a Student’s *t*-test was used to identify significant differences between means of cell types (Additional file 4). All data are illustrated as the mean ± standard error. Analyses were conducted using GraphPad Prism 5 software (GraphPad Software Inc.).

## Supplementary Information

**Additional file 1: Dataset S1.** Mapping rates of Trinity assembly.

**Additional file 2: Figure S1.** Zeiss laser capture microdissection experiment. (A) Cell population separated by laser and (B) areole tissue remaining after cell population extracted.

**Additional file 3: Dataset S2.** Leaf stage cluster annotation, median expression values, GO term counts and GO enrichment results.

**Additional file 4: Figure S2.** qRT-PCR validation of leaf stages and cell types experiments. All copy numbers of probed genes were normalized by copy numbers of α-tubulin.

**Additional file 5: Dataset S3**. NPCD and PCD cell cluster annotation, median expression values, GO term counts, and GO enrichment results.

**Additional file 6: Dataset S6.** qRT-PCR primer information.

## Data Availability

Raw sequencing data files are available in the NCBI SRA database with project accession NO. PRJNA591467. This Transcriptome Shotgun Assembly project has been deposited at DDBJ/ENA/GenBank under the accession NO. GJFM00000000. The version described in this paper is the first version, GJFM01000000.

## Abbreviations

DEG: Differentially expressed gene
BP: Base pair
GO: Gene Ontology
nt: Nucleotide
FPKM: Fragments per kilobase of exon model per million mapped reads
RNA-Seq: RNA sequencing
PCD: Programmed cell death
NPCD: Non-PCD
ROS: Reactive oxygen species
qRT-PCR: Quantitative reverse transcriptase polymerase chain reaction
