# Supplementary figures and images for: RNA-Seq analysis reveals potential regulators of programmed cell death and leaf remodelling in lace plant (Aponogeton madagascariensis)
Source: BMC Plant Biol. 2021 Aug 13;21:375. doi: 10.1186/s12870-021-03066-7 (PMC8361799; doi:10.1186/s12870-021-03066-7)

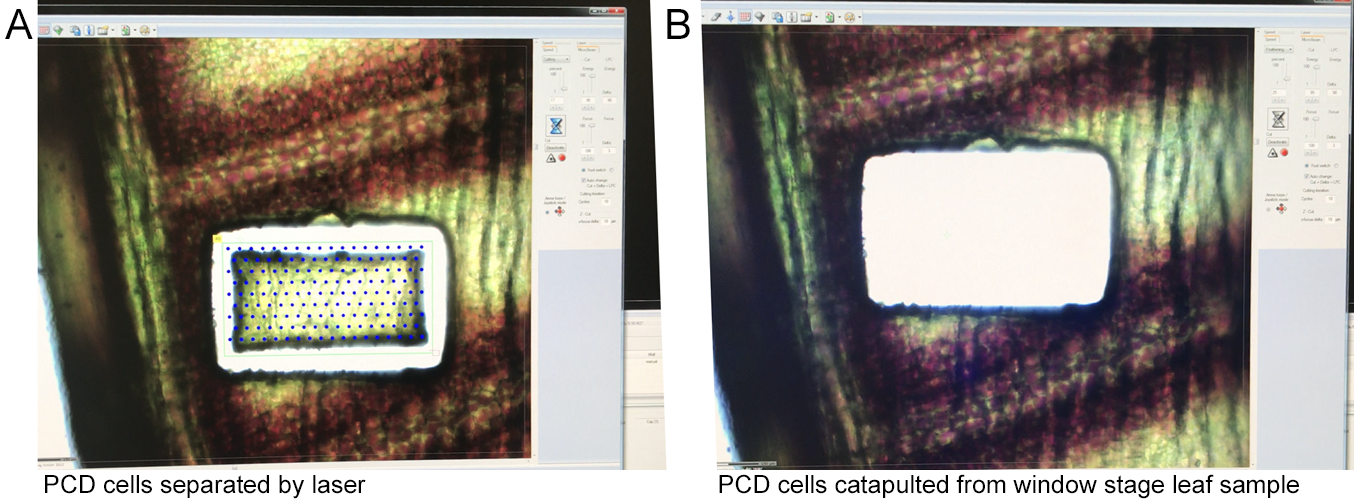

Supplement: Supplementary file 2 — Additional file 2: Figure S1. Zeiss laser capture microdissection experiment. (A) Cell population separated by laser and (B) areole tissue remaining after cell population extracted. [file 12870_2021_3066_MOESM2_ESM.tif]

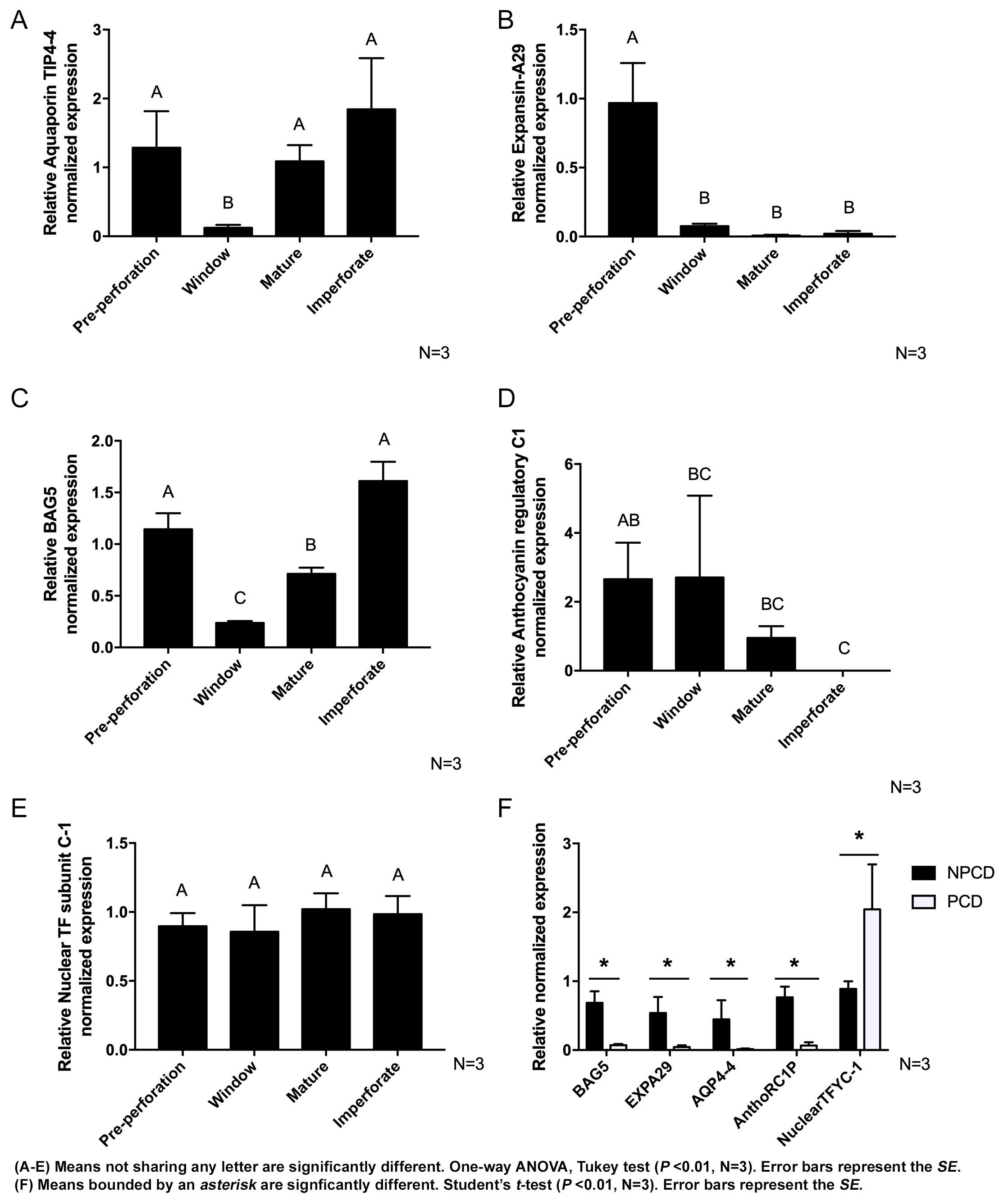

Supplement: Supplementary file 4 — Additional file 4: Figure S2. qRT-PCR validation of leaf stages and cell types experiments. All copy numbers of probed genes were normalized by copy numbers of α-tubulin. [file 12870_2021_3066_MOESM4_ESM.tif]
